# Stronger Tests of Mechanisms Underlying Geographic Gradients of Biodiversity: Insights from the Dimensionality of Biodiversity

**DOI:** 10.1371/journal.pone.0056853

**Published:** 2013-02-25

**Authors:** Richard D. Stevens, J. Sebastián Tello, María Mercedes Gavilanez

**Affiliations:** 1 Department of Biological Sciences, Louisiana State University, Baton Rouge, Louisiana, United States of America; 2 Center for Conservation and Sustainable Development, Missouri Botanical Garden, St. Louis, Missouri, United States of America; 3 Museo de Zoología, Pontificia Universidad Católica del Ecuador, Pichincha, Quito, Ecuador; University of Western Ontario, Canada

## Abstract

Inference involving diversity gradients typically is gathered by mechanistic tests involving single dimensions of biodiversity such as species richness. Nonetheless, because traits such as geographic range size, trophic status or phenotypic characteristics are tied to a particular species, mechanistic effects driving broad diversity patterns should manifest across numerous dimensions of biodiversity. We develop an approach of stronger inference based on numerous dimensions of biodiversity and apply it to evaluate one such putative mechanism: the mid-domain effect (MDE). Species composition of 10,000-km^2^ grid cells was determined by overlaying geographic range maps of 133 noctilionoid bat taxa. We determined empirical diversity gradients in the Neotropics by calculating species richness and three indices each of phylogenetic, functional and phenetic diversity for each grid cell. We also created 1,000 simulated gradients of each examined metric of biodiversity based on a MDE model to estimate patterns expected if species distributions were randomly placed within the Neotropics. For each simulation run, we regressed the observed gradient onto the MDE-expected gradient. If a MDE drives empirical gradients, then coefficients of determination from such an analysis should be high, the intercept no different from zero and the slope no different than unity. Species richness gradients predicted by the MDE fit empirical patterns. The MDE produced strong spatially structured gradients of taxonomic, phylogenetic, functional and phenetic diversity. Nonetheless, expected values generated from the MDE for most dimensions of biodiversity exhibited poor fit to most empirical patterns. The MDE cannot account for most empirical patterns of biodiversity. Fuller understanding of latitudinal gradients will come from simultaneous examination of relative effects of random, environmental and historical mechanisms to better understand distribution and abundance of the current biota.

## Introduction

Geographic gradients in biodiversity are ubiquitous across space and time [1], the tree of life [2] and different dimensions of biodiversity [3], [4], [5]. Despite demonstration of the ubiquity of such gradients, numerous explanatory mechanisms have accumulated with none achieving hegemony [2]. Proposed mechanisms can be divided into three main groups. Contemporary environmental conditions, in particular gradients of temperature, seasonality, productivity and energy have been perhaps the most frequently explored mechanisms and are proposed by many to be the primary determinants of contemporary patterns of diversity [6], [7], [8], [9]. Although contemporary climate may act as a strong form of selection, ultimately differences in diversity are due either to differences in speciation or extinction rates operating through millennia [10]. Accordingly, recently developed historical explanations have quickly and positively influenced thinking about diversity gradients [11]. Differential rates of speciation and extinction predicted under metabolic theory [12], variation in amount of time for speciation [13] and tropical niche conservatism [12], [14] have provided powerful alternative directions that have rapidly expedited understanding of the formation of diversity gradients, especially those related to latitude [15]. Lastly, better understanding of stochastic processes such as the mid-domain effect (MDE) [16], [17] has been instrumental in demonstrating how simply randomly placing species geographic ranges within a bounded domain (e.g., a continent) can create relatively strong gradients in species richness associated with latitude and longitude [18], as well as with underlying environmental gradients [19]. Determination of the degree to which simple stochastic mechanisms are related to empirical biological patterns is of fundamental importance. Moreover, these more parsimonious explanations should be definitively vetted before more complex explanations are considered [17].

In the original inception of the MDE [16], models were introduced to demonstrate the types of species richness gradients (i.e. shape and magnitude of relationship) that could be produced given the random placement of species geographic ranges. These models demonstrated that randomizing the position of species geographic ranges within a bounded domain could produce relatively strong latitudinal richness gradients. The implicit hypothesis underlying such models is that random distribution of species within bounded domains is responsible for the production of diversity gradients, a proposition that dramatically contrasts with the widespread notion that environmental gradients play a strong role in the development of species richness patterns. Tests of the MDE have now been conducted for numerous taxa, across most continents, and overall there is moderate to substantive agreement between gradients in species richness produced by the MDE and those that occur in nature ([18], but also see [20]).

The last 19 years that MDE models have been in use have also witnessed a large conceptual shift in their application [18]. In its original formulation, the MDE was presented as a null model to test the hypothesis that quantitative characteristics of empirical diversity gradients were no different than those generated by randomly placing species geographic ranges within latitudinal bounds [16], [17]. Recently, however, focus has shifted to explicitly consider that the MDE is not just a pattern generating null model used to contrast contemporary patterns of species richness, but an important causative mechanism underlying these very gradients [18], [21]. Nonetheless, as with all putative explanatory mechanisms, fit between observed and expected levels of diversity is necessary but not sufficient evidence to infer causation. Indeed, mechanism is hard to definitively demonstrate in macroecology, in particular because vast spatial or temporal extents preclude manipulative experimentation based on our physical capabilities and ethical considerations. Testing single predictions based on correlates of empirical patterns represents only a weak test of mechanism [22], [23].

One possibility to improve inference of mechanisms underlying macroecological patterns is the simultaneous examination of multiple patterns predicted by a particular mechanism under consideration. This idea has contributed to a number of more rigorous approaches to infer mechanism from pattern such as: a) the concept of exchangeability [22], b) pattern oriented modeling [24], and c) the “dipswitch” test [25]. All of these approaches stress the fact that if a mechanism is the true explanation for a complex system (such as a biodiversity gradient), effects should extend to multiple patterns in nature.

Although patterns of species richness have been the focus of ample research on geographical ecology, biodiversity represents a complex system that is multifaceted and includes much more than variation in species richness [26]. Functional, genetic, phylogenetic and phenetic variation [27] represent only a few of the many dimensions of biodiversity that exhibit strong geographic gradients [28], [29] and are related to important ecological processes [30]. Effects of mechanisms underlying geographic gradients of species richness may extend to other dimensions of biodiversity and such an extension may provide for a powerful launching point for inference of the mechanistic basis of broad scale diversity gradients. Here we use the dimensionality of biodiversity to develop a more powerful test to investigate the effects of one of the more prominent yet controversial mechanisms purported to drive diversity gradients at global scales, the mid-domain effect.

Examining mechanistic effects across numerous dimensions of biodiversity can provide stronger inference. For example, the mechanism underlying the MDE hypothesis is the random placement of species ranges within a constrained domain. Such a mechanism can result in spatial structure of species richness produced by the overlap of geographic ranges [18]. Nonetheless, the MDE hypothesis does not claim that traits of species are randomly distributed across phylogeny, that species ecological function is unrelated to phenotype, or that the randomization of species distributions implies the randomization of other characteristics of species. Although MDE was originally formulated to address patterns of species richness, through extension it directly applies to other dimensions of biodiversity. This is because tied to each geographic range are all of the phenotypic characteristics of species that contribute to other dimensions of biodiversity. Through sampling effects, variation across sampling sites in the number of overlapping geographic ranges will result in not only changes in the number of species but also changes in all other dimensions of biodiversity that are related to which species (i.e. the identity) are involved. This is not to say that MDE predicts a peak in phylogenetic, functional, or phenetic diversity in the middle of the domain, per se, like for species richness. Rather, through this process, MDE will create gradients in other dimensions of biodiversity. Thus, given the empirical relationships between traits, phylogeny and function and hypothesizing that the MDE is an important determinant of observed biodiversity gradients, then the random distribution of species within a domain should be able to account not only for gradients in species richness, but for gradients in other forms of biodiversity as well. Deviations from the empirical patterns would suggest that indeed the distribution of species is not random, and that other environmental or historical processes are responsible for the position of species geographic ranges and the gradients in biodiversity that result from their overlap. Such a multidimensional test is a more rigorous means to evaluate if species are randomly distributed within a domain and whether a stochastic process could possibly have generated contemporary gradients in biodiversity.

Despite the rigor involved in examining dimensionality of biodiversity and different yet associated patterns generated from a particular mechanism across numerous dimensions, this approach has been used rarely in macroecology and never to investigate the MDE. Herein, we begin by examining if a mid-domain effect generating latitudinal gradients in species richness indeed extends to generate gradients in phylogenetic, functional and phenetic dimensions of biodiversity. Then, we use this hypothesis to conduct a more rigorous test of the MDE. If the MDE is an important mechanism generating contemporary gradients of biodiversity then expected values based on such a model should exhibit significant goodness of fit to empirical gradients.

## Methods

We focused on the New World super-family Noctilionoidea (*sensu stricto*—Noctilionidae+Mormoopidae+Phyllostomidae) [31], in particular because this superfamily is monophyletic [32], species rich [33], [34], and phenotypically diverse [35], making it an ideal group for large-scale analyses of biodiversity. Moreover, a well-resolved phylogeny exists that characterizes evolutionary relationships among most extant species [32]. Patterns of diversity were characterized based on range maps for 133 species obtained from Patterson *et al.*, [36]. The continental New World was divided into 10,000-km^2^ grid cells (100×100 km) and those species whose distribution overlapped a particular cell were included in the list of species for that cell. Richness was calculated as the number of species whose distributions overlapped each cell. All cells in the New World not occupied by any species were not considered in analyses.

For each grid cell we estimated three different aspects of phylogenetic diversity. We used the topology and branch lengths from the noctilionoid portion of a mammal super tree [37]. Based on this phylogeny, we calculated Faith's phylogenetic diversity measure (PD) [38], phylogenetic species variability (PSV) [39] and phylogenetic species clustering (PSC) [39]. PD is perhaps the oldest and most widely used measure of phylogenetic diversity. Despite the fact that phylogenetic diversity is strongly correlated with species richness, including this measure could provide comparative insights because of such wide historical use. PSC and PSV have been more recently developed to describe phylogenetic community structure [39]. When measured for an assemblage, PSV characterizes the degree of relatedness among taxa across the entire phylogeny; it is directly proportional to the average pair-wise distance among species in a phylogeny. In contrast, PSC is a measure of how clustered species are at the tips of the tree; it is directly proportional to nearest neighbor distance among taxa based on a phylogeny. The supertree was manipulated in R using the package APE [40] and all measures of phylogenetic diversity were calculated for each grid cell using the R package PICANTE [41].

We used distribution of trophic guild membership to estimate functional diversity of species assemblages within grid cells. We categorized species into six functional groups based on diet: aerial insectivores, frugivores, gleaning animalivores (those that glean vertebrates and invertebrates from surfaces such a leaves, tree trunks and the ground [3]), nectarivores, piscivores or sanguinivores. We then counted the number of species per functional group within each grid cell. From these data (i.e., number of species per functional group), we then determined richness of functional groups, functional diversity based on Shannon's index [42], and functional evenness based on Camargo's index [43]. Other methods have been developed to characterize functional diversity, in particular use of functional dendrograms [44]. Moreover, while the above scheme of examining distribution of species across trophic guilds has been successfully used to characterize the functional diversity of bats [3], other schemes have also been proposed [45], [46]. We did not use these but instead use a diet-based classification because the most important and geographically consistent functional characteristic of bat species is their diet [47] and dietary differences directly reflect differences in how bats contribute to ecosystem processes such as energy flow and nutrient cycling [48]. We were specifically interested in diversity of the explicit pathways whereby bats move carbon and energy through ecosystems (e.g., frugivory, insectivory, sanguinivory). Moreover, the only other examination of large-scale patterns of functional diversity in bats [3] is based on such a scheme and this greatly facilitates comparisons with other findings.

We characterized the phenetic diversity of assemblages within grid cells based on 7 morphological measures [49]: forearm length, greatest length of skull, condylobasal length, length of maxillary toothrow, breadth of post-orbital constriction, breadth of braincase, breadth across upper molars. Measures were based on the mean of at least 4 males and 4 females of most species. Through allometry, morphological variables are correlated with each other. Accounting for such correlations might be important if comparisons were made across morphological variables. Nonetheless, we calculated indices by combining measures across species and used these to compare assemblages across geography. Since correlations are relative to morphological characteristics and not to species or geography they have no influence on the difference between patterns generated by the MDE and those across empirical gradients. Although these measures estimate the size and shape of the trophic apparatus, namely the cranium [49], [4], they are strongly related to interspecific variation in overall size. We log-transformed values for each morphological measure and then estimated three measures of phenotypic diversity for each grid cell based on those species in the cell. Morphological volume was estimated as the product of the ranges of all morphological variables. Morphological variability was estimated by the standard deviation (STD) of the lengths of a minimum spanning tree uniting all species in multidimensional space. Overall degree of proximity was estimated as average distance between a particular species and its nearest morphological neighbor.

We compared empirical gradients of diversity to those generated by a MDE model in which species distributions were randomly placed within a bounded domain. We defined the domain as the latitudinal bounds of the distribution of extant species of Noctilionoidea. These represent soft boundaries [50] based on limits to physiological tolerances in response in particular to temperature [51] or other environmental characteristics that constrains the empirical distribution of the clade. Ideally, domains are defined by limits of biogeographic units. Nonetheless, limits of noctilionoids cut across numerous ecoregions and biomes in both North and South America making such biogeographic unit delimitation unfeasible. Arbitrary domain delineation could serve to predispose a MDE [52], [53]. This was not found to be the case here.

In our MDE simulation model, only the distribution of species within the domain was randomized while phylogenetic, functional, and phenetic characteristics of each species were retained. Dimensions of diversity are correlated with one another in these data as well as in general [3], [4], [54], [55]. This is primarily because different dimensions are simply different reflections of the same phenotype, given that phenotype is associated with phylogenetic position, and because, due to a sampling effect, areas with higher richness will tend to have higher values of multiple measures of diversity. Moreover, these inter-correlations likely contribute to some degree to similarity of gradients across dimensions. Thus, we retained this biological reality in our randomization model. To produce a random geographic distribution for a species, one cell within the domain was randomly selected. Starting at this cell, the range was allowed to grow stochastically by sending “dispersers” from each occupied cell to a randomly selected adjacent cell. This process continued until the number of occupied cells in the stochastic distribution was identical to the number of cells occupied by the empirical distribution of the species (this is similar to a spreading dye algorithm) [56]. This was done for all species. Species lists for all cells within the domain were generated, and measures of biodiversity for each cell estimated. This was repeated 1,000 times to generate a distribution of diversity gradients for each measure of biodiversity expected under the MDE hypothesis.

For each iteration of the MDE model, we conducted simple linear regression [57] whereby expected values were independent variables and observed values were dependent variables. Often correspondence between empirical diversity and that predicted by the MDE is evaluated with a coefficient of determination (r^2^, summarized in [18]). Nonetheless, such a measure of goodness of fit is necessary but not sufficient to demonstrate direct correspondence between empirical and randomized data, and under some circumstances can be misleading [56], [58]. In particular, any monotonic relationship between observed and expected results is predisposed to have a high r^2^ even when the relationship is nonlinear. Although not independent from r^2^, evaluating the slope and intercept of the regression line can provide further information regarding correspondence because dependent and independent variables are expressed in the same units. A slope different than 1 could indicate systematic deviations along a gradient and an intercept different from zero could indicate an additive difference between observed and expected values; both indicate a lack of fit between the MDE expectations and empirical diversity gradients. We conducted stronger tests by considering goodness of fit from both perspectives [20]. Coefficient of determination (r^2^) was used to characterize amount of variation in empirical patterns accounted for by MDE whereas slopes and intercepts were used as quantitative measures of goodness of fit.

We retained estimates of r^2^, intercept, and slope and generated distributions of these parameters based on 1,000 iterations of the MDE model. If there is good correspondence between expected and observed values then the central tendency of r^2^ should be high; we used this as an illustrative indicator. As a quantitative test we used the bi-variate distribution of slopes and intercepts. The distribution of intercepts should reflect a parametric mean of 0 and the distribution of slopes should reflect a parametric mean of one. Individual tests of slope and intercept are not statistically independent. Thus, as a single test for significant differences between observed and simulated diversity gradients, we constructed bivariate 95% confidence envelopes around the distribution of slopes and intercepts from simulation analyses by assuming a bivariate normal distribution and drawing a 95% probability contour ellipse. If confidence envelopes did not overlap one and zero, respectively, then we concluded that these distributions reflect some other relationship and do not indicate correspondence between empirical diversity values and those expected given the MDE.

## Results

**Figure 1:**
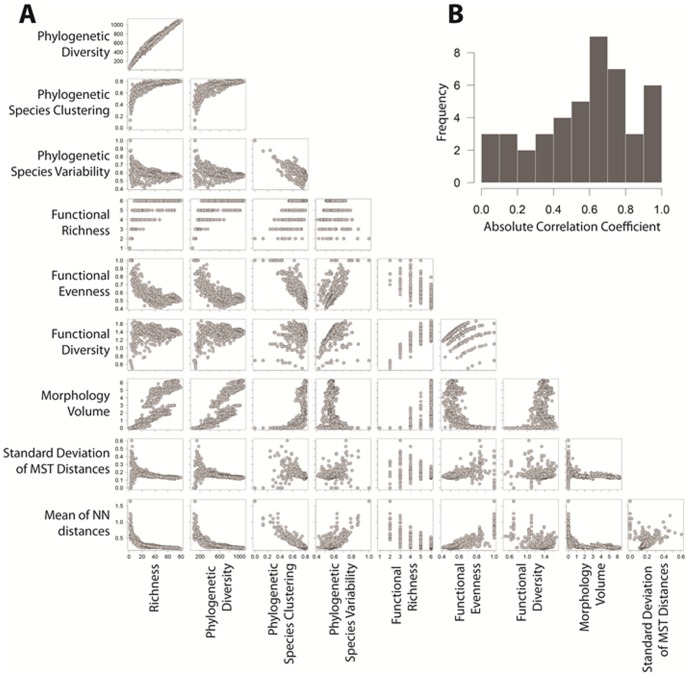
Pairwise associations among indices of biodiversity (A) and histogram describing the magnitudes of Spearman correlation coefficients (B).

**Figure 2:**
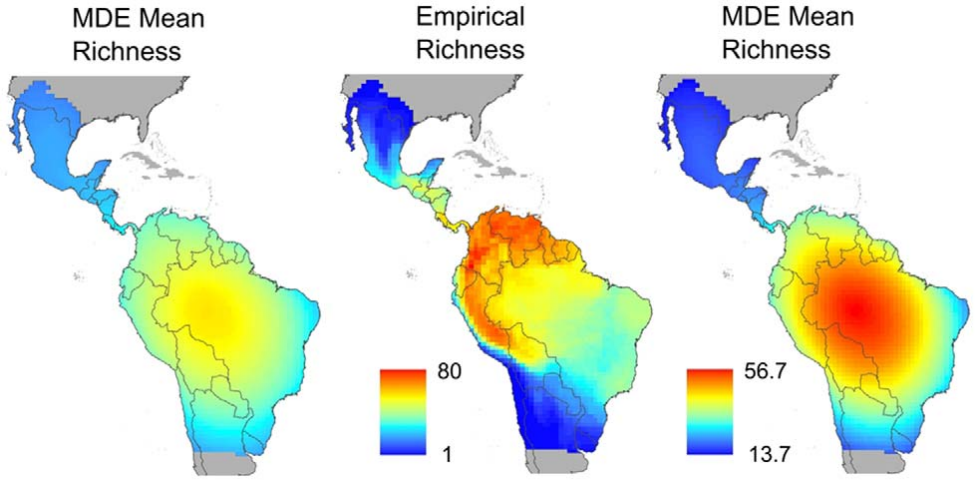
Empirical (center) and MDE-generated (left and right) spatial variation in species richness of Noctilionoidea in the New World. Left and right panels present unscaled and scaled spatial variation, respectively. Unscaled mean simulated gradients correspond to MDE generated variation that is scaled the same as for empirical patterns. Scaled mean simulated gradients correspond to MDE generated variation that ranges according to the magnitude of MDE results. Red shades depict areas of high species richness whereas blue shades represent areas of low species richness. Colors in the left and middle columns are directly comparable. Colors in the right column are not comparable to those of the two left columns because they are scaled differently. Areas in grey are those occurring outside the geographic distribution of the Noctilionoidea.

**Figure 3:**
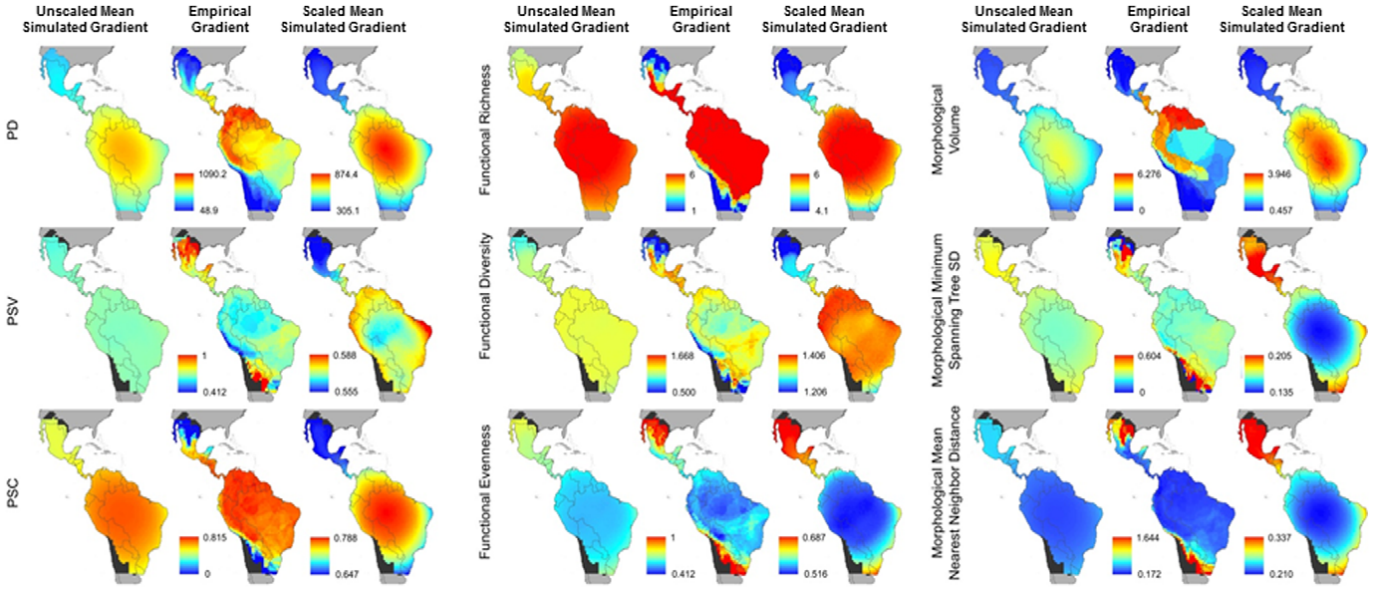
Spatial variation in phylogenetic (PD, PSV, PSC), functional (functional richness, functional diversity, functional evenness), and phenetic (morphological volume, minimum spanning tree SD, mean nearest neighbor distance) components of biodiversity of Noctilionoidea. Each characteristic is represented by three panels. Unscaled mean simulated gradients correspond to MDE generated variation that is scaled the same as for empirical patterns (left). Empirical patterns are in the middle panel. Scaled mean simulated gradients correspond to MDE generated variation that ranges according to the magnitude of MDE results (right). Colors in the left and middle columns are directly comparable. Colors in the right column are not comparable to columns on the left because they are scaled differently. Areas in grey are those occurring outside the geographic distribution of the Noctilionoidea. Black areas are those for which diversity measures could not be estimated because they registered only one species.

Indices of biodiversity exhibited varying degrees of correlation (Fig. 1), with PD, morphological volume, functional richness and functional evenness exhibiting the greatest correlations with species richness. Indices were variable exhibiting both low and high inter-correlations. The average absolute pairwise Spearman's correlation coefficient was 0.58. Strong spatial gradients exist for all measured indices of biodiversity (Fig. 2, Fig. 3). In particular, indices reflecting numbers of items (e.g., species richness, phylogenetic diversity, functional richness, functional diversity, morphological volume) were greatest at tropical latitudes and lowest at the highest latitudes. Measures reflecting the equitability of items (e.g., PSV, functional evenness, STD of minimum spanning tree based on morphology, mean nearest-neighbor distance based on morphology) exhibited weaker gradients. These measures decreased toward the equator and indicated greater species packing in terms of phylogeny and morphology and increased dominance in terms of particular feeding guilds.

### The MDE on patterns of species richness

The MDE algorithm imposed on the geographic distribution of Noctilionoidea produced gradients in species richness that are similar to empirical gradients as well as those of prior MDE analyses of all New World bats [17] and the Noctilionoidea [59], [60]. For both empirical and MDE gradients, species richness was greatest toward the equator, least toward the highest latitudes, and changed fairly monotonically across this range (Fig. 2, right panel).

**Figure 4:**
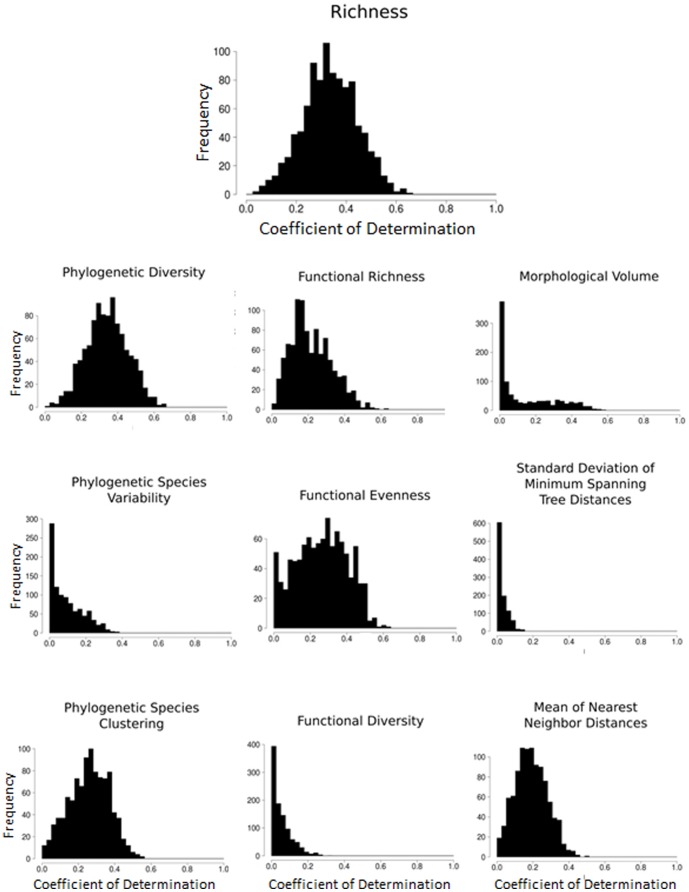
Histograms describing goodness of fit based on coefficients of determination (r^2^) between empirical and MDE-generated variation in species richness across 1,000 runs of the MDE model.

**Figure 5:**
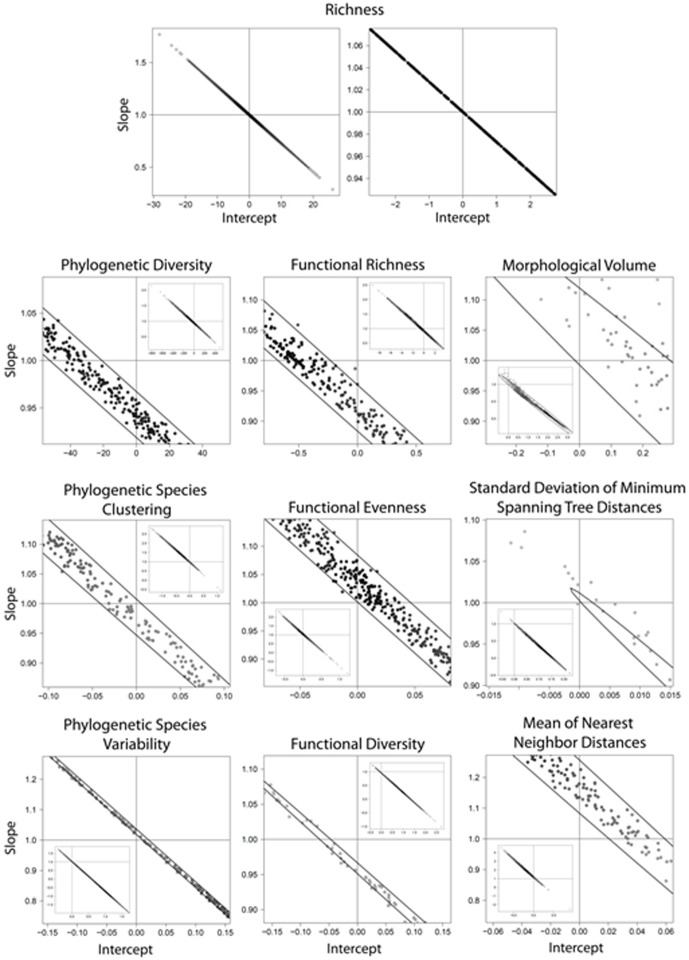
Scatter plots indicating variation in combinations of slope and intercept for the relationship between empirical and simulated gradients in different measures of biodiversity generated from the MDE analysis. Dots represent the combination of slope and intercept for each run. Large figures represent a subset of simulation runs that were close to the theoretical expectation of zero intercept and slope of unity. Inset represents relationship based on all 1,000 simulation runs. The two intersecting lines represent the theoretical expectation. If there is good fit between observed and expected values then the confidence envelope will overlap the point of zero intercept and slope of unity.

Considerable statistical correspondence existed between empirical patterns of species richness and those expected based on the MDE (Figs. 4 and 5). Expected values on average accounted for approximately 34% of the variation among cells in observed species richness (Fig. 4, Table S1). Average intercept was −0.25 and average slope was 1.01. The bivariate confidence envelope overlapped 1 for slopes and zero for intercepts suggesting good correspondence between empirical and MDE generated gradients of species richness. Results suggest that the slope and intercept of the empirical gradient fall within those expected based on MDE.

### The MDE on other dimensions of biodiversity

In general, random placement of species geographic ranges within the domain of Noctilionoidea generates monotonic latitudinal gradients in expected values of all other dimensions of biodiversity (Fig. 3). In particular, values characteristic of high species richness, for example high degree of phylogenetic clustering, large number of functional groups or a high degree of species packing, were predicted by the MDE to be of high magnitude at the equator and monotonically decrease toward the limits of the geographic distribution of the Noctilionoidea. Even dimensions exhibiting low empirical correlations with species richness exhibited strong latitudinal gradients expected by the MDE. These simulations substantiate that a mechanism that can create strong gradients in species richness can also generate strong gradients in other dimensions of biodiversity. There were two notable exceptions involving individual indices, in particular PSV and FD. Despite strong spatial gradients, both measures did not demonstrate peaks in values towards the equator. Instead, intermediate values existed in the middle of the distribution of Noctilionoidea.

#### Phylogenetic diversity

Deviations between expected and empirical patterns were substantive for aspects of phylogenetic diversity (Figs. 4 and 5). Coefficients of determination between observed and expected values were typically close to zero for PSV and were moderate (26% and 34%, respectively) for PSC and Faith's PD (Fig. 4, Table S1). Mean intercepts and slopes of the relationship between observed and expected phylogenetic diversity values significantly diverged from 0 and 1, respectively (Table S1). In fact, for PSV and PSC, the expected intercept of zero and the expected slope of one fell outside of the MDE distribution.

#### Functional diversity

Patterns of functional diversity produced by the MDE were weaker but exhibited similar patterns to those of empirical gradients (Fig. 3). Only modest agreement between MDE and empirical gradients of functional diversity was quantitatively indicated by regression analyses (Table S1, Figs. 4 and 5). Average amount of variation in empirical values of functional diversity accounted for by the MDE ranged from 6 to 25%. The 95% confidence envelope around the bivariate distribution of slopes and intercepts did not overlap expected values of 0 and 1, respectively, indicating not only a significant additive effect (indicated by deviation in intercept) whereby the magnitude of diversity is on average different across grid cells, but that significantly different rate of change (indicated by deviation in slope) reflects different spatial gradients as well.

#### Phenetic diversity

Deviations between expected and empirical patterns were greatest for aspects of phenetic diversity (Fig. 3). Coefficients of variation between observed and expected values were low for all three measures (Table S1, Fig. 4). Expected slopes and intercepts for two of three measures fell well outside of the distribution of those obtained from simulations (Fig. 5).

## Discussion

The multifaceted nature of biodiversity provides a framework for more robust insights into the mechanistic basis of latitudinal gradients. Different dimensions exhibit similar latitudinal gradients suggesting that a common mechanism may drive the ubiquity of the pattern. One starting point is examination of the MDE. Indeed, the MDE generated spatial gradients in all measured indices of biodiversity and in most cases a peak resulted in the middle of the domain at the equator. To this end, effects of MDE are extendable across dimensions of biodiversity. Moreover, assessing the degree to which the MDE fits empirical gradients in dimensions of biodiversity other than species richness can provide for a more rigorous test of its mechanistic effect.

Patterns of species richness based on range overlaps of New World bats are similar to expected patterns based on other MDE models [17], [59], [60]. Nonetheless, much poorer fit of expected values to empirical values of other dimensions indicate the superficial degree to which the MDE accounts for gradients of biodiversity. Moreover, a seemingly serendipitous fit of model expectations to patterns of species richness and not to other dimensions of biodiversity further brings into question the operation of geometric constraints as a primary mechanism driving global biodiversity gradients.

### Agreement of mid-domain model with species richness gradients

Overall magnitude of species richness across grid cells for both real data and those generated by the MDE model were similar as reflected by the zero intercept of the relationship. Moreover, rate of change across grid cells was also similar as reflected by a slope of 1. Amount of variation in empirical data accounted for by those expected based on the MDE was relatively low (mean r^2^ = 0.34). Such modest explained variation is hard to interpret in terms of the efficacy of the MDE as a mechanism generating diversity gradients. From the perspective of ecological work in general, accounting for 34 percent of the variation in the data given a model is substantive, if not impressive. This is much higher than values of r^2^ reported to be typically reached in ecological work (0.025–0.054, [61]). In terms of accounting for geographic gradients in species richness however, many contemporary environmental variables account for more. Across the 393 analyses examined by Field *et al.* [8], the primary environmental predictor of species richness accounted for approximately 54% of variation among sites in terms of numbers of species. Moreover, argument has been made that the expected amount of variation accounted for in empirical values of species richness by the MDE should be close to 100% [20]. Because of the mathematical relationship between the slope and r^2^, a high r^2^ is expected from a unity slope. This may be particularly true for the MDE because randomizing the same number of geographic ranges characterized by the same size distribution should yield a distribution of species richness across cells that is similar to the empirical distribution [20]. Thus, similar variances and an expected slope of unity should naturally generate a high r^2^. The MDE model employed here accounted for nowhere near 100% of the variation and was less predictive than many other commonly studied environmental predictors [8]. To this end, even for patterns of species richness where the MDE seems to perform best, there is still much variation to be accounted for.

### Other dimensions of biodiversity

When species composition of grid cells was determined by an MDE model, all but two characteristics of biodiversity (PSV, FD) exhibited marked mid-domain peaks in magnitude. Our simulations demonstrated that if patterns of biodiversity are the product of the MDE not only will a species richness gradient result but corresponding MDE gradients of other dimensions of biodiversity will result as well. Despite reasonable correspondence between empirical species richness values and those expected based on a MDE model, correspondence with other dimensions of biodiversity was considerably less and quite variable. In general, aspects of phylogenetic diversity fit best, aspects of phenetic diversity fit worst and aspects of functional diversity fit to an intermediate degree.

MDE effects can also be organized around measures of quantity and measures of distribution. Those measures sensitive to the number of things, for example species richness, phylogenetic diversity, functional richness and size of the morphological volume exhibited empirical patterns that were most related to predictions from a MDE, albeit only weakly. In contrast, those characteristics that measure how entities (i.e., species) are distributed in reference to each other (e.g., PSV, PSC, STD-MST distances, mean NND) tended to exhibit the least agreement with MDE predictions (range of r^2^ = 0.028–0.26).

Biodiversity metrics that reflect numbers of things such as PD, species and functional richness lack resolution as to species-specific ecological characteristics that embody variation in biodiversity and thus may be predisposed to covary in a simple way latitudinally, in particular varying from high at the equator to low at high latitudes. Richness very coarsely describes the magnitude of number of things while ignoring ecological differences that are important characteristics of biodiversity. Moreover, the same richness value can be obtained by many combinations of species with very different characteristics. In contrast, of all components examined, morphology is perhaps the most ecologically descriptive and the characteristic most aligned with particular properties of the niche [62]. Characteristics that are sensitive to species differences exhibit more fine-scale variation and thus a unique value results from a unique set of species even if that set reflects the same richness.

One criticism of MDE models is that they do a poor job of accounting for empirical patterns of beta-diversity [50]. A concept firmly underlying beta-diversity is differences in species composition given the same species richness [63]. One likely reason that the MDE exhibits poorer fit to other dimensions of biodiversity may be that attributes of those dimensions may be important determinants of beta-diversity along gradients. Environmental characteristics such as climate or biotic interactions form important filters that limit the kinds of species co-occurring at a particular place [64]. Moreover, spatial variation in such environmental characteristics creates beta-diversity representing the non-random selection of species that co-occur because of these filters. One reason why MDE models perform poorly in terms of capturing salient patterns of beta-diversity [50] may be that they do not capture complex functional, phylogenetic and morphological characteristics of species that are important in local assembly and thus are insensitive to many of the characteristics of species important to patterns of turnover.

### Serendipity and strong inference from multiple dimensions of biodiversity

Based on consideration of functional, phenetic and phylogenetic characteristics, the MDE accounts for little spatial variation of biodiversity given its dimensionality. The limited fit of MDE expectations to empirical latitudinal gradients in species richness may represent only a serendipitous association. This may be particularly true in the New World where the greatest spatial dimension is latitudinal. The greatest spatial dimension for most other major mechanisms (i.e., productivity, seasonality) proposed to account for diversity gradients also is latitudinal in the New World [9]. Given the collinearity of MDE with many other environmental drivers such as energy, climate and seasonality, the modest fit of the MDE to empirical richness gradients should be suspect, especially because it accounts for less variation than other contemporary mechanisms [9], [60]. New World bats exhibit strong latitudinal gradients in species richness [17], [33] that are strongly related to environmental [9] and historical [14] phenomena that vary primarily in a latitudinal direction in the New World. Only the weakest predictions of the MDE are upheld for empirical patterns based on our analyses, namely a peak in species richness at the middle and a monotonic decrease toward the edge of the domain. Such agreement between empirical and expected variation in species richness may only represent serendipity based on collinearity with other putative mechanisms in the New World [20].

Examination of multiple patterns of biodiversity as employed here provides stronger inference regarding the mechanistic basis of latitudinal diversity gradients. Our approach is not the only means of testing if manifestations of MDE are extendable. The MDE makes explicit predictions regarding beta-diversity and the distribution of geographic range endpoints that are not upheld [50], [52]. The MDE also makes predictions regarding latitudinal change in the shape of the frequency distribution of range sizes [65] that have yet to be tested empirically. Thus one advantage of the MDE is that it provides many predictions that allow strong inference [20]. Nonetheless, many of these predictions apply only to the MDE and thus cannot be used as a general battery to test other mechanisms proposed for geographic gradients of diversity. In contrast, tests involving the dimensionality of biodiversity can be applied to any mechanism. Indeed, if a particular mechanism causes a richness gradient and effects are general, then they are likely to manifest across other dimensions of biodiversity as well.

The approach used here has provided for a much stronger test of the MDE. Moreover, other mechanisms still need to be evaluated using this stronger approach making it still unclear whether any of the proposed mechanisms can attain hegemony. Even if a single mechanism achieves hegemony, it is likely that no one mechanism operates in isolation. More recent approaches examining the relative abilities of different mechanisms to account for variation in species richness represent an improvement because they can be used to indicate the most likely candidate [8], [9]. An additional innovation has been the development of process-based models that mechanistically employ different hypotheses into the same model and use this to generate predicted gradients with which to contrast those found in nature [66], [67], [68]. Nonetheless, these approaches have not addressed how effects of processes extend to a number of dimensions of biodiversity. Future work should go beyond testing single mechanisms such as was done here for MDE, and explore the relative ability of competing mechanism to account for the dimensionality of gradients of biodiversity. Different dimensions of biodiversity could easily be incorporated into process-based models [68] to generate even more comprehensive understanding of interactive effects. Moreover, examination of the dimensionality of biodiversity could also greatly improve inference in terms of importance of multiple competing mechanisms. Typically success of a particular mechanism has been assessed by the amount of variation in species richness accounted for or, even better, the amount of unique variation accounted for. Nonetheless, success could also be judged regarding the relative ability of mechanisms to account for patterns across the phenotype of biodiversity (i.e. how extendable their effects are) whereby mechanisms that account for variation across more dimensions are deemed more important than those accounting for variation across only a few. Indeed, better comprehension of interactive effects among mechanisms will greatly enhance our understanding of the formation of biodiversity gradients. Future research should compete numerous mechanisms based on multiple predictions across numerous dimensions of biodiversity to improve understanding of drivers of these conspicuous and important global patterns.

## Supporting Information

Table S1 **Summary statistics for coefficient of determination (r^2^), intercept and slope of relationships between empirical values of biodiversity and those generated from a MDE.** 95% C. I. and 99% C. I. refer to the 95 and 99 percent confidence intervals, respectively, around the distributions of intercepts and slopes.
(DOCX)
