# Supplementary material for: Stronger Tests of Mechanisms Underlying Geographic Gradients of Biodiversity: Insights from the Dimensionality of Biodiversity
Source: PLoS One. 2013 Feb 25;8(2):e56853. doi: 10.1371/journal.pone.0056853 (PMC3581556; doi:10.1371/journal.pone.0056853)
Supplement: Supplementary file 1 [file pone.0056853.s001.docx]

Dimension of Biodiversity	Measure of Diversity	Mean r2	Intercept Mean	95% C. I.			99% C. I.			Slope Mean	95% C. I.			99% C. I.		
Taxonomic	Species Richness	0.335	-0.248	-0.726	to	0.229	-0.876	to	0.380	1.007	0.994	to	1.020	0.990	to	1.024
Phylogenetic	Diversity	0.341	-66.475	-76.228	to	-56.722	-79.302	to	-53.649	1.047	1.031	to	1.062	1.027	to	1.067
	Species Variability	0.097	0.671	0.644	to	0.699	0.636	to	0.707	-0.143	-0.191	to	-0.096	-0.206	to	-0.081
	Species Clustering	0.259	-0.367	-0.385	to	-0.349	-0.391	to	-0.343	1.436	1.438	to	1.487	1.431	to	1.494
Functional	Richness	0.218	-1.232	-1.369	to	-1.095	-1.412	to	-1.052	1.135	1.112	to	1.159	1.104	to	1.166
	Diversity	0.055	0.832	0.804	to	0.861	0.795	to	0.869	0.356	0.336	to	0.377	0.329	to	0.383
	Evenness	0.263	-0.004	-0.018	to	0.010	-0.022	to	0.015	1.049	1.024	to	1.075	1.016	to	1.083
Phenetic	Volume	0.144	1.524	1.473	to	1.575	1.457	to	1.591	0.349	0.325	to	0.372	0.318	to	0.380
	Standard Deviation of MST Distances	0.028	0.104	0.101	to	0.106	0.100	to	0.107	0.330	0.312	to	0.347	0.307	to	0.353
	Mean of Nearest Neighbor Distances	0.190	-0.208	-0.219	to	-0.197	-0.223	to	-0.193	2.040	1.993	to	2.087	1.979	to	2.102
